# Adhesion of pancreatic tumor cell clusters by desmosomal molecules enhances early liver metastases formation

**DOI:** 10.1038/s41598-024-68493-6

**Published:** 2024-08-06

**Authors:** Niclas Dietrich, Ramon Castellanos-Martinez, Julia Kemmling, Arnd Heuser, Michael Schnoor, Camilla Schinner, Volker Spindler

**Affiliations:** 1 Department of Biomedicine, University of Basel, Basel, Switzerland; 2 Department of Molecular Biomedicine, CINVESTAV-IPN, Mexico, Mexico; 3 Institute of Anatomy and Experimental Morphology, University Medical Center Hamburg Eppendorf, Martinistrasse 52, 20246 Hamburg, Germany; 4 Animal Phenotyping Platform, Max-Delbrück Center for Molecular Medicine in the Helmholtz Association, Berlin, Germany; 5 Hannover Medical School, Institute of Applied and Functional Anatomy, Carl-Neuberg-Straße 1, 30625 Hannover, Germany

**Keywords:** Cancer models, Metastasis, Pancreatic cancer, Cell adhesion, Desmosomes

## Abstract

Desmosomes are intercellular adhesion complexes providing mechanical coupling and tissue integrity. Previously, a correlation of desmosomal molecule expression with invasion and metastasis formation in several tumor entities was described together with a relevance for circulating tumor cell cluster formation. Here, we investigated the contribution of the desmosomal core adhesion molecule desmoglein-2 (DSG2) to the initial steps of liver metastasis formation by pancreatic cancer cells using a novel ex vivo liver perfusion mouse model. We applied the pancreatic ductal adenocarcinoma cell line AsPC-1 with and without a knockout (KO) of DSG2 and generated mouse lines with a hepatocyte-specific KO of the known interacting partners of DSG2 (DSG2 and desmocollin-2). Liver perfusion with DSG2 KO AsPC-1 cells led to smaller circulating cell clusters and a reduced number of cells adhering to murine livers compared to control cells. While this was independent of the expression levels of desmosomal adhesion molecules in hepatocytes, we show that increased cluster size of cancer cells, which correlates with stronger cell–cell adhesion and expression of desmosomal molecules, is a major factor contributing to the early phase of metastatic spreading. In conclusion, impaired desmosomal adhesion results in reduced circulating cell cluster size, which is relevant for seeding and attachment of metastatic cells to the liver.

## Introduction

Desmosomes are intercellular adhesion complexes which mediate the structural integrity of all epithelial tissues and are especially important in tissues that experience mechanical stress. The cadherin-type transmembrane adhesion molecules desmoglein (DSG) 1–4 and desmocollin (DSC) 1–3 form the desmosomal core and facilitate binding to the counterparts on adjacent cells in a Ca^2+^-dependent manner. Plakoglobin (PG) and plakophilins (PKPs) are members of the armadillo family of proteins linking the cytoplasmic tails of the cadherins with desmoplakin (DP) which serves to anchor intermediate filaments^1,2^. Desmosomes are not static complexes, but undergo continuous remodeling and may switch between calcium-dependent and calcium-independent states. This may allow fine-tuning of cell–cell adhesion and adaption to changing external forces^3,4^. The principal relevance of desmosomes is highlighted by the diseases caused by desmosomal dysfunction. This includes the blistering skin disease pemphigus and the hereditary disorder Arrhythmogenic Cardiomyopathy. Here, either autoantibodies against the desmosomal cadherins DSG1 and DSG3 lead to blistering of the skin and mucosa^5^ or mutations in desmosomal molecules result in arrhythmias, cardiac fibrosis, and impaired cardiac function^6,7^.

Moreover, contribution of desmosomal molecules to carcinogenesis and metastasis formation has been described previously^8–10^. The formation of metastases is a multistep cascade, including the resolution of cell–cell and cell–matrix adhesion at the primary site, invasion and migration into the surrounding tissue, intravasation, dissemination through blood or lymph, extravasation into distant organs and formation of new metastases^11,12^. Within this cascade, desmosomal molecules have been associated with specific steps and can act as tumor promotors or suppressors depending on the cellular context^13,14^. For example, the desmosomal adhesion molecule DSG2 is downregulated in primary colon cancer, which correlates with poor survival^15^. In contrast, it is upregulated in non-small cell lung cancer with a protective effect of DSG2 depletion in a xenograft mouse model^16^. In pancreatic cancer cells, loss of DSG2 promoted a pro-migratory behavior, which was dependent on ERK signaling and expression of PG^17^.

For formation of metastases, the relevance of circulating tumor cells (CTCs) and CTC clusters have become evident in the past decade. CTCs and CTC clusters arise from primary or metastatic tumors after entering the circulation via lymph or blood vessels as either a solitary cell or a cluster^18,19^. CTCs and CTC clusters were detected in most cancer entities, such as breast, lung, colorectal and pancreatic cancer and are associated with a poor prognosis^20–24^. While CTC clusters are less frequent than single CTCs, CTC clusters show an increased potential to form metastases in animal models. Importantly, expression of the desmosomal plaque protein PG was shown to contribute to clustering of CTCs and subsequent metastasis^20^. Together, these data outline a general contribution of desmosomal molecules to invasion and metastasis formation. However, the relevance of desmosomal proteins to certain steps of the metastatic cascade is only partially understood.

In this study, we focused on pancreatic cancer, as it is one of the most lethal cancers with an overall 5-year survival rate of 9%^25^. One major survival limiting step during the pathological evolution of this cancer entity is the metastatic spread to the liver as the next capillary bed of the portal vein circulation. As up to now only very limited therapeutic options are available, we need a better understanding of the steps leading to pancreatic cancer liver metastasis formation. Here, previous data of our group indicate a contribution of the desmosomal adhesion molecule DSG2, the main isoform expressed in pancreatic epithelial cells, to tumor cell migration and invasion of pancreatic carcinoma cells^17^. Clinically, high expression of DSG2 correlates with significantly reduced survival rates in patients (data available from Human Protein Atlas, version 23.0, https://www.proteinatlas.org/ENSG00000046604-DSG2/pathology/pancreatic+cancer), suggesting a relevant contribution of this adhesion protein to the pathological cascade of pancreatic cancer.

Based on this, we here aim to investigate the role of DSG2 expression during the initial phase of metastatic spreading and attachment of cancer cells in distant organs and tissue. To investigate the major spreading route to the liver, we established an ex vivo liver perfusion mouse model and tested the relevance of desmosomal adhesion molecules in tumor cells compared to hepatocytes with respect to seeding to the liver. We employed the pancreatic ductal adenocarcinoma cell line AsPC-1 bearing a KO of DSG2 and generated hepatocyte-specific Dsg2 and Dsc2 KO mouse models. Our data show that expression of DSG2 is relevant for sufficient cell–cell adhesion and formation of larger cancer cell clusters, which are more likely to attach to liver tissue in perfusion experiments. Importantly, this process was independent from hepatic expression of, and thus probably binding to, desmosomal adhesion molecules. In summary, this study highlights the relevance of DSG2 and desmosomal function for pancreatic tumor cell cluster formation and the initial phase of metastatic spreading to the liver.

## Results

### Establishment of an ex vivo liver perfusion mouse model to mimic the initial steps of metastatic spreading to the liver

**Figure 1:**
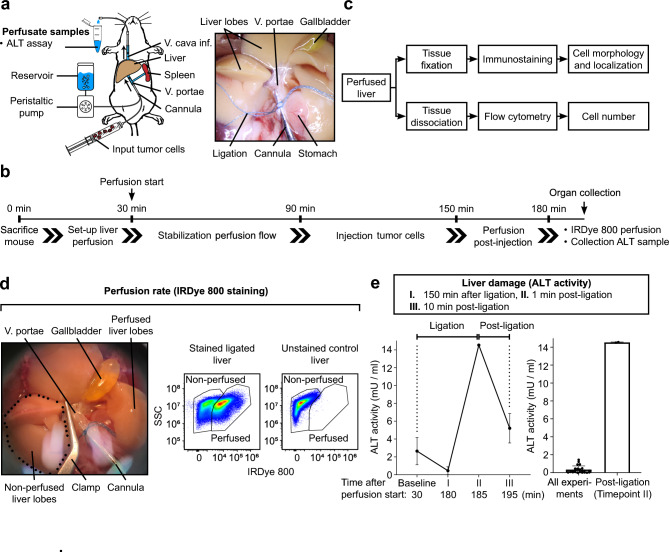
Set-up of the murine liver perfusion model. (**a–c**) Schematic and representative image of the liver perfusion mouse model with corresponding timeline of the experiments and analysis pipeline. (**d**) Validation experiment for the IRDye 800-perfusion method to determine the percentage of sufficiently perfused liver tissue (perfusion rate). As control condition, the right hepatic branch of the portal vein was ligated during perfusion with the IRDye 800 indicated in the image on the left and liver tissue was dissociated and subjected to flow cytometry analysis. Results are presented as scatter blot with side scatter area (SSC-A) vs. IRDye 800 intensity. An unstained liver served as control to set the “non-perfused” gate. For all liver perfusion experiments included in the following figures, perfusion rate attained minimum 90%. (**e**) Validation experiment for quantification of liver cell damage via the enzymatic activity of alanine aminotransferase (ALT). Output samples were collected at the start of the experiment (baseline), 150 min after ligation (180 min after perfusion start) of the right branch of the hepatic portal vein (I), 1 min (II) and 10 min after re-opening of the ligation (III). ALT activity in all liver perfusion experiments included in the following figures were in the range of baseline levels confirming sufficient perfusion during the experiments.

To study the role of desmosomal proteins in the initial phase of metastasis spread to the liver, we established an ex vivo perfusion mouse model. Livers were perfused in situ via cannulation of the portal vein and connected to an extracorporeal circulation. Via this route, fluorescently labeled cancer cells were perfused into the mouse liver under controlled conditions. Additional cannulation of the inferior vena cava was performed to obtain the perfusate (outflow) after passing through the liver (Fig. 1a). After perfusion, the morphology, localization and number of cancer cells attached to the liver tissue was determined by histological work up including immunostaining and flow cytometry analysis of dissociated liver tissue (Fig. 1b,c). In addition, the injected cell solution (input) was analyzed via flow cytometry (Supplementary Fig. S1).

For our set-up, labeled tumor cells were injected after cannulation and stabilization of the perfusion flow for a total of 90 min. To avoid clotting, cells were injected at a low concentration over a period of 60 min. Subsequently, non-attached cells were washed out for 30 min (Fig. 1b). To control for sufficient perfusion of each liver, the percentage of perfused liver tissue was determined by addition of a fluorescent dye (IRDye 800) during the final perfusion phase subsequent to tumor cell injection to label all cells (tumor cells and liver tissue) in contact with the perfusion buffer. This quality control approach was validated in a control experiment where the right branch of the portal vein was ligated to cut-off single liver lobes from perfusion. After perfusion and tissue dissociation, the non-perfused versus perfused tissue was detectable in flow cytometry analysis compared to a control liver without addition of IRDye 800 (Fig. 1d). To additionally monitor damage of the liver tissue due to insufficient perfusion, alanine aminotransferase (ALT) activity was determined in the perfusate during the final experimental phase. ALT is a cytosolic and mitochondrial enzyme of hepatocytes and is released upon cell damage. In respective control experiments, ligation of the right branch of the hepatic portal vein with insufficient perfusion of the dependent lobes led to an increased enzymatic activity of ALT in the output perfusate after re-opening of the ligation (Fig. 1e). Importantly, the ALT levels after ligation highly exceeded the ALT levels detected in all regular perfusion experiments, which were similar to baseline levels directly after start of perfusion (Fig. 1e). Together, these validation experiments confirm the quantification of labeled liver cells and ALT activity in the perfusate as suitable parameters to control for sufficient liver perfusion and were applied for all subsequent experiments. All liver perfusion experiments exhibited a percentage of IRDye 800 labled liver cells > 90% and ALT activity less than 2 mU/mL.

### Deficiency of DSG2 in AsPC-1 cells leads to reduced hepatic cell attachment

**Figure 2:**
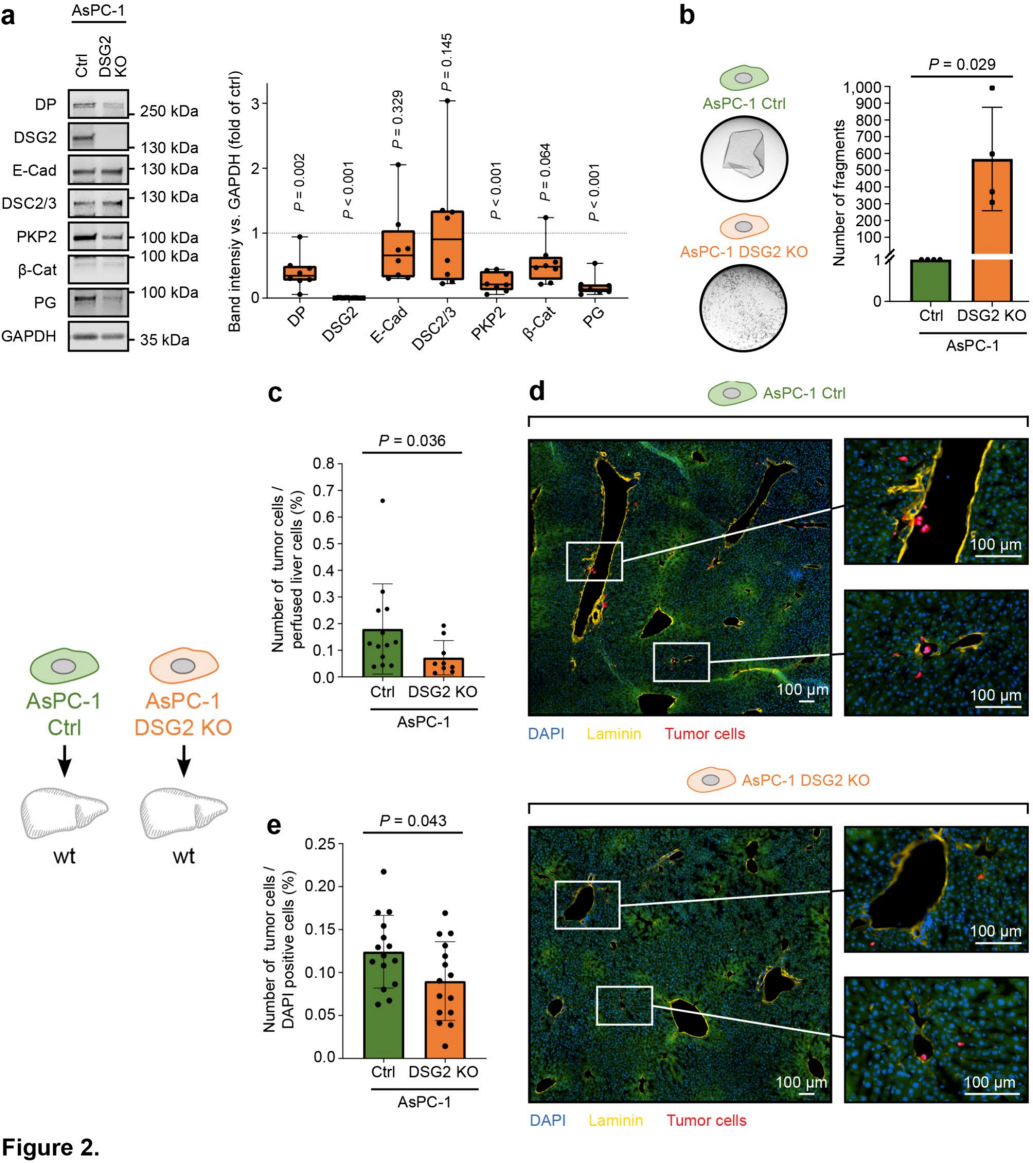
DSG2 KO in AsPC-1 cells leads to loss of cell–cell adhesion and reduced attachment in perfused livers. (**a**) Representative Western blot analysis on the left with corresponding densitometric analysis on the right of the desmosomal proteins desmoplakin (DP), desmoglein-2 (DSG2), desmocollin-2/3 (DSC2/3), plakophilin-2 (PKP2), plakoglobin (PG) and the adherens junctions molecules E-cadherin (E-Cad), and β-catenin (β-Cat) in AsPC-1 control (Ctrl) and DSG2 knockout (KO) cells. GAPDH served as loading control. n = respective mean of 8 independent biological replicates, two-tailed unpaired t test. (**b**) Dissociation assay of AsPC-1 control and DSG2 KO cells. Corresponding images of monolayer fragmentation after application of defined mechanical stress are depicted on the left side. n = respective mean of 4 independent biological replicates, two-tailed Mann–Whitney test. (**c–e**) Ex vivo liver perfusion of AsPC-1 control and DSG2 KO cells into wild-type (wt) mouse livers as depicted in the scheme on the left. The number of tumor cells per perfused liver cells was determined using flow cytometry in (**c**) as outlined in Fig. 1. n = 13 (control) and 9 (DSG2 KO) perfused livers, two-tailed Mann–Whitney test. (**d**) Representative immunostainings of perfused liver sections. Sections were stained with laminin to label vessel walls (yellow), DAPI to visualize cell nuclei (blue) and CellTrace Far Red (CTFR, red) marks perfused AsPC-1 tumor cells. The green background is a result of the autofluorescence of the liver tissue. Magnified areas show the local invasion of AsPC-1 tumor cells into the liver tissue. (**e**) Corresponding analysis of stained liver sections to determine the number of attached cells per total cell number determined via DAPI nuclear staining. n = 5 perfused livers, mean of 2 consecutive sections per transversal level (top, middle, bottom of the lobe), two-tailed unpaired t test.

After establishment of the liver perfusion set-up, we investigated the influence of DSG2 expression on the attachment of CTCs in liver tissue. As tumor model, we chose the pancreatic ductal adenocarcinoma cell line AsPC-1, which forms liver metastases after orthotopic implantation in mice^26,27^ and exhibits DSG2-dependent migratory and invasive behavior^17^. We generated a DSG2 knockout (KO) line from parental AsPC-1 using CRISPR/Cas9 and confirmed the KO by sequencing and Western blot analysis (Fig. 2a). Basic characterization of the junctional components suggested a disruption of the desmosomal complex with reduced protein levels of DP, PKP2, and PG in DSG2-deficient cells, while in contrast the adherens junction proteins E-Cadherin and β-Catenin were not significantly altered. To analyze the functional impact of these findings, cell–cell adhesion was determined by cell dissociation assays. Here, fragmentation of a detached confluent cell monolayer after application of a defined mechanical stress was quantified. DSG2 KO resulted in fragmentation of the cell layer while control layers stayed intact, demonstrating reduced adhesive interactions with neighboring cells (Fig. 2b). However, in line with persistent expression of other adhesion molecules, small cell–cell clusters were still detectable under DSG2 KO conditions. To determine the relevance of DSG2 expression for early metastatic attachment of pancreatic tumor cells in the liver, AsPC-1 control and DSG2 KO cells were dissociated and perfused into wild-type (wt) mouse livers. The amount of metastatic attachment was quantified by flow cytometry and calculated as ratio of attached tumor cells per perfused liver cells (Fig. 2c, Supplementary Fig. S1). Here, perfusion with DSG2-deficient AsPC-1 cells led to a reduced number of tumor cells attached to liver tissue compared to AsPC-1 control cells. Visualization of sections of perfused livers revealed attachment of AsPC-1 cells mainly near portal fields and within the liver sinusoids and analysis confirmed reduced deposition of DSG2 KO cells (Fig. 2d,e). These data indicate that loss of the adhesion molecule DSG2 disrupts the desmosomal complex with a reduction in cell–cell adhesion and decrease of the rate of liver attachment of perfused tumor cells.

### Hepatic expression of Dsg2 or Dsc2 does not affect cancer cell attachment

**Figure 3:**
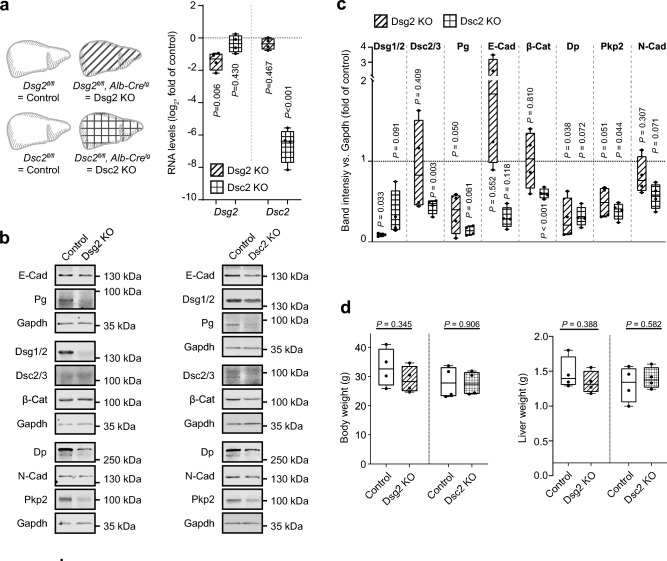
Characterization of hepatocyte-specific Dsg2 and Dsc2 KO mouse models. (**a**) On the left: scheme of applied Dsg2 (*Dsg2*^*fl/fl*^*; Alb-Cre*^*tg*^) and Dsc2 (*Dsc2*^*fl/fl *^;* Alb-Cre*^*tg*^) knockout (KO) mouse liver samples. *Dsg2*^*fl/fl*^ or *Dsc2*^*fl/fl*^ mice, respectively, served as control. Striped bars indicate Dsg2 KO, squared bars Dsc2 KO. On the right: confirmation of respective mRNA depletion by qPCR analysis. Values were normalized to Gapdh house keeper gene. n = 4 mice, two-tailed unpaired t test. (**b**) Representative Western blot images and (**c**) corresponding band intensity analysis of the junctional components E-cadherin (E-Cad), plakoglobin (Pg), desmoglein-1/2 (Dsg1/2), desmocollin-2/3 (Dsc2/3), β-catenin (β-Cat), desmoplakin (Dp), N-cadherin (N-Cad) and plakophilin-2 (Pkp2) in Dsg2 and Dsc2 KO mouse liver samples. Gapdh served as loading control. n = 4 mice, Dsg2 KO liver vs. control liver: Dsg1/2: two-tailed unpaired t test with Welch’s correction, all other: two-tailed unpaired t test. Dsc2 KO liver vs. control liver: Dsc2/3, β-Cat and N-Cad: two-tailed unpaired t test, all other: two-tailed unpaired t test with Welch’s correction. (**d**) Body and liver weight of Dsg2 KO and Dsc2 KO mice compared to the corresponding control mice. n = 4 mice, two-tailed unpaired t test.

To analyze in more detail the mechanisms underlying reduced attachment of DSG2 KO cells, we tested whether desmosomal binding partners of DSG2 are required on hepatocytes. Based on the known binding of DSG2 to either DSGs or DSCs, we generated liver-specific KO mouse models deficient for Dsg2 or Dsc2, the two isoforms of desmosomal proteins expressed in hepatocytes. We bred transgenic mice homozygous for *LoxP*-flanked (floxed) *Dsg2* or *Dsc2* with animals expressing the Cre recombinase under the hepatocyte-specific albumin promotor (Alb-Cre), resulting in either a loss of Dsg2 (*Dsg2*^*fl/fl*^*; Alb-Cre*^*Ttg*^*,* Dsg2 KO) or Dsc2 (*Dsc2*^*fl/fl*^*; Alb-Cre*^*tg*^, Dsc2 KO) in hepatocytes. The efficiency of the depletion was confirmed by qPCR and Western blot analysis of liver tissue (Fig. 3a–c). We performed a detailed characterization of these models, with analysis of the junctional protein expression, which revealed disruption of the hepatic desmosomal complex by loss of Dsg2 or Dsc2, respectively. Western blot analysis showed reduced levels of the desmosomal plaque proteins Pg, Pkp2, and Dp in response to depletion of Dsg2, while levels of the adherens junction proteins E- and N-cadherin, and β-catenin (β-Cat) were not affected. In contrast, hepatic deficiency of Dsc2 resulted in addition to decreased protein levels of β-Cat. No differences were observed for the body and liver weight of the transgenic mice (Fig. 3d) and no other overt phenotype was detectable, indicating that Dsg2 and Dsc2 together with proper desmosomal composition are dispensable for liver function under homeostatic conditions.

**Figure 4:**
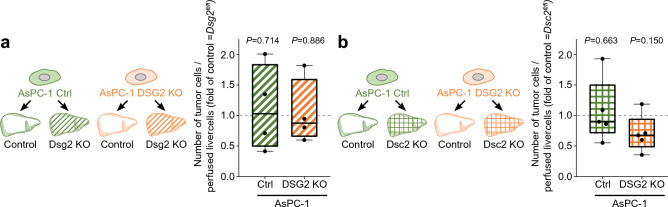
Hepatic deletion of Dsg2 or Dsc2 is not affecting metastatic cell attachment. (**a, b**) Depict the results of mouse perfusion experiments with injection of AsPC-1 control (Ctrl) or DSG2 knockout (KO) cells into the livers of transgenic mice with either a hepatic KO of Dsg2 (*Dsg2*^*fl/fl*^*; Alb-Cre*^*tg*^, Dsg2 KO*)* or Dsc2 (*Dsc2*^*fl/fl*^*; Alb-Cre*^*tg*^, Dsc2 KO*)*. *Dsg2*^*fl/fl*^ or *Dsc2*^*fl/fl*^ mice, respectively, served as control. Values of KO livers were normalized to the respective control livers. Striped bars represent Dsg2 KO livers and squared bars Dsc2 KO livers. The number of tumor cells per perfused liver cells was determined using flow cytometry. (**a**) AsPC-1 control n = 6 (Control) and 4 (Dsg2 KO) livers, AsPC-1 DSG2 KO n = 4 (Control) and 4 (Dsg2 KO) livers, both two-tailed Mann–Whitney test vs. respective control liver. (**b**) AsPC-1 control n = 4 (Control) and 5 (Dsc2 KO) livers, two-tailed unpaired t test with Welch’s correction vs. respective control liver. AsPC-1 DSG2 KO n = 4 (Control) and 5 (Dsc2 KO) livers, two-tailed unpaired t test vs. respective control liver.

After establishing these models**,** liver perfusion experiments were performed to investigate the relevance of Dsg2 or Dsc2 expression in the liver tissue for metastasis attachment. To do so, we perfused AsPC-1 control and DSG2 KO cells into mouse livers with deficiency for Dsg2 or Dsc2 (Fig. 4a,b). Perfusion of transgenic *Dsg2*^*fl/fl*^ or *Dsc2fl/fl* livers, respectively, without Cre expression served as controls. Quantification of the number of tumor cells attached to liver tissue using flow cytometry revealed no significant difference in KO versus control livers for both animal models and was further independent from expression of DSG2 on the tumor cells. This suggests that the increased attachment rate of DSG2 expressing tumor cells is independent from the hepatic expression of the classical binding partners Dsg2 or Dsc2.

### Deficiency of DSG2 results in reduced clustering of AsPC-1 cells

**Figure 5:**
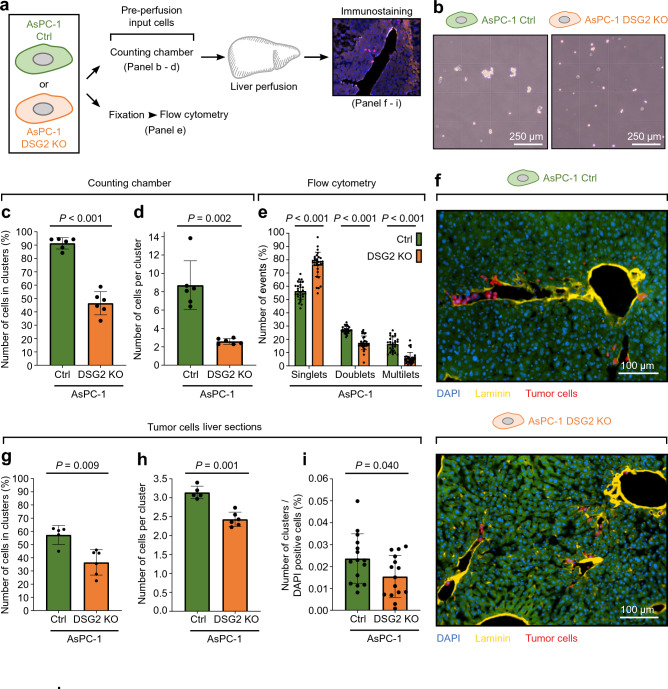
Reduced cell clustering in AsPC-1 DSG2 KO cells. (**a**) Schematic representation of experimental set-up. (**b**) Representative images of AsPC-1 control (Ctrl) and DSG2 knockout (KO) input tumor cells in counting chambers. Images were taken during the cell preparation for liver perfusion experiments in Fig. 2. (**c–e**) Quantification of cell clustering of AsPC-1 control and DSG2 KO input tumor cells via image analysis of the counting chambers and flow cytometry. Counting chamber (**c**, **d**): n = 6 independent cell preparations. Cells in clusters: two-tailed unpaired t test. Cells per cluster: two-tailed unpaired t test with Welch’s correction. Flow cytometry analysis (**e**): n = 18 (Ctrl) and 14 (DSG2 KO) independent cell preparations, two-way RM ANOVA, same seeding matched, Geisser-Greenhouse correction, Sidak’s post hoc test. (**f**) Stained sections of wild-type (wt) mouse livers after perfusion experiments with AsPC-1 control or DSG2 KO cells. Representative immunostainings of laminin to label the hepatic vessel wall (yellow), DAPI to mark cell nuclei (blue), and CellTrace Far Red (CTFR, red) to label perfused AsPC-1 tumor cells are depicted on the right side. The green background results from autofluorescence of the liver tissue. (**g–j**) Corresponding analysis of the clustering behavior and number of attached clusters and cells within the perfused liver is depicted. For the discrimination of a single cell and a cell cluster, a cell–cell distance of 50 µm and a minimal number of two cells were required to fulfill the criteria of a cell cluster. Percentage of cells in cluster: n = 5 (Ctrl) and 6 (DSG2 KO) livers, two-tailed Mann–Whitney test. Cells per cluster: n = 5 (control) and 6 (DSG2 KO) livers, two-tailed unpaired t test. Clusters per cells: n = 5 perfused livers, mean of 2 consecutive sections per transversal level (top, middle, bottom of the liver), two-tailed unpaired t test.

Based on the previous experiments, DSG2-mediated binding of tumor cells to hepatocytes appears to be unlikely to contribute to the reduced retainment of DSG2 KO AsPC-1 in the liver. We thus focused on the phenotype and behavior of the perfused tumor cells based on previous studies of CTC clusters, which demonstrated a contribution of cluster size to metastasis formation^28^. We analyzed the data acquired during the liver perfusion experiments with respect to the cluster phenotype prior to perfusion and after deposition in the liver (Fig. 5a). Analysis of cluster morphology of detached AsPC-1 cells prior to perfusion (input cells) revealed that the AsPC-1 control cells form larger clusters with multiple cells, while DSG2 KO cells almost entirely remained as single cells or small clusters (Fig. 5b). We observed that 91% of the AsPC-1 control cells and only 46% of the DSG2 KO cells were part of a cell cluster, defined as accumulation of two or more cells (Fig. 5c). Additionally, the number of cells per cluster was significantly higher in AsPC-1 control cells compared to the DSG2 KO cells (Fig. 5d). This was substantiated by flow cytometry analysis of the input tumor cells which displayed enhanced clustering of detached AsPC-1 control cells even after respective sample processing including fixation and cell straining (Fig. 5e, Supplementary Fig. S1). Here, we detected an increased percentage of cell doublets and multilets and a reduced percentage of singlets in AsPC-1 control cells compared to the DSG2 KO cells. After analyzing cell clustering of the input cells, we evaluated cluster morphology of perfused tumor cells attached in the liver (Fig. 5f). Investigating WT liver sections after perfusion revealed more clusters with a higher percentage of cells in clusters and an increased number of cells per cluster in the AsPC-1 control cells compared to the DSG2 KO cells (Fig. 5g–i). This enhanced clustering and our finding of a higher rate of AsPC-1 control cells attached to liver tissue suggest that the clustering of pancreatic cancer cells plays a role during the early phase of liver deposition and metastatic seeding.

### Cluster size correlates with hepatic tumor cell deposition

**Figure 6:**
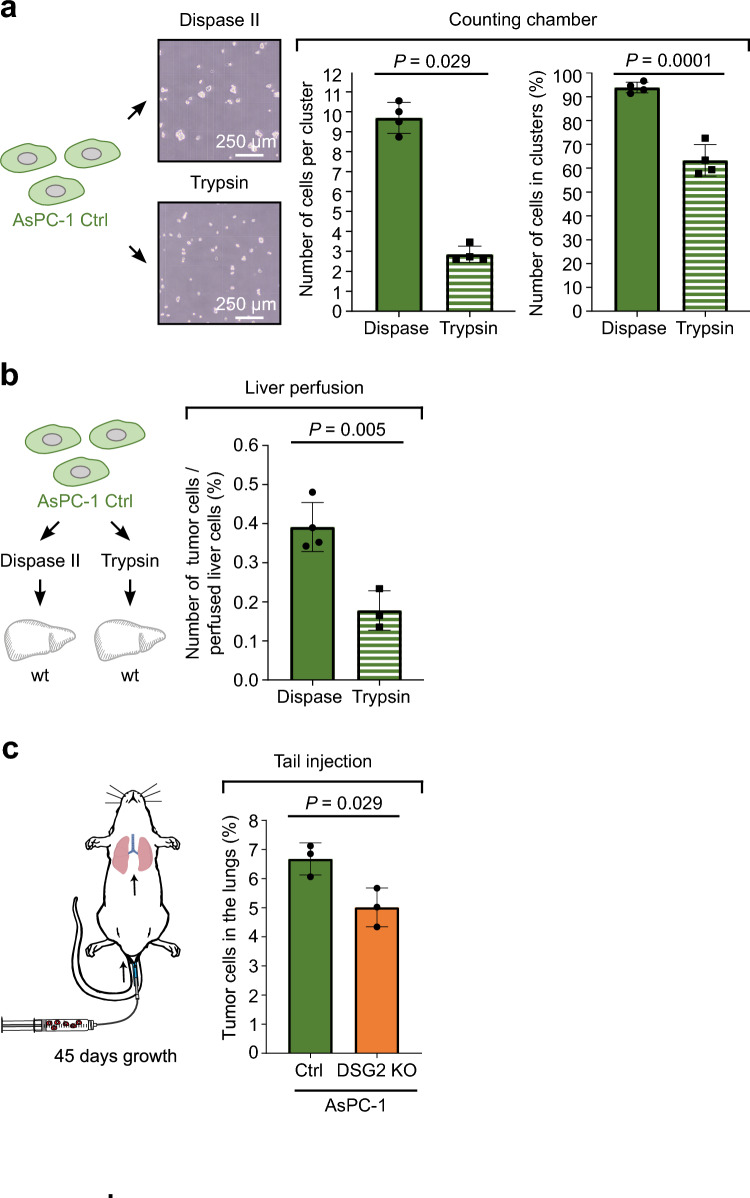
Reduced cancer cell cluster size correlated with reduced attachment in liver perfusion experiments. (**a**) Quantification of clustering of AsPC-1 control (Ctrl) cells by image analysis of the counting chambers after being detached with either dispase II or trypsin. Both bar graphs: n = 4 independent cell preparations. Cells in clusters: two-tailed unpaired t test. Cluster size: two-tailed Mann–Whitney test. (**b**) Mouse perfusion experiments with wild-type (wt) mouse livers and AsPC-1 control cells after detachment of cells with either dispase II or trypsin. The number of tumor cells per perfused liver cell was determined using flow cytometry. n = 4 (dispase II) and 3 (trypsin) livers, two-tailed unpaired t test. (**c**) In vivo tail vein injection of AsPC-1 GFP^+^ cells as depicted on the left. After 45 days of metastasis growth, the lungs were dissociated and the percentage of GFP^+^ cancer cells was analyzed by flow cytometry. n = 3 mice per group, two-tailed unpaired t test.

To further investigate the importance of cell cluster size for early formation of liver metastases, AsPC-1 control cells were dissociated with different enzymes (Fig. 6a). Here, detachment via trypsin led to a significant reduction in cell cluster size (mean of approx. 3 cells vs. 9 cells) and a decrease in the percentage of cells in clusters (mean of 63% vs. 94%) compared to cell detachment with dispase II, as performed for the previous perfusion experiments. Having established a method to reduce cluster size of AsPC-1 cells, wt livers were perfused with dissociated AsPC-1 control cells (Fig. 6b). To mainly focus on cluster size, cells were fixed after dissociation and prior to perfusion to exclude an effect of surface protein digestion and degradation. In these experiments, AsPC-1 control cells dissociated with trypsin and thus reduced cluster size led to a lower number of tumor cells attached to perfused livers compared to cells detached with dispase and larger cell cluster size. These experiments indicate that cell cluster size is relevant for the deposition of pancreatic cancer cells to the liver. Finally, to gain more insight into the in vivo relevance of this finding and for subsequent steps of metastasis formation and growth, we injected AsPC-1 cells intravenously and analyzed tumor cell growth in the next capillary bed, in this case the lung, 45 days post-injection (Fig. 6c). Here, DSG2 KO cells led to lower metastatic tumor cell burden. This supports the hypothesis that reduced initial attachment of DSG2 KO cells translates into reduced metastasis formation which is in line with increased survival of patients with low DSG2-expressing pancreatic tumors.

## Discussion

In this study, we established an ex vivo liver perfusion mouse model, which allowed us to investigate the early phase of metastatic spread of tumor cells to the liver by orthotopic perfusion under controlled conditions. We showed that loss of DSG2 in AsPC-1 cells leads to reduced cell–cell adhesion, smaller cell clusters and a lower tumor cell deposition in perfused livers. By creating transgenic mouse models with either a hepatic KO for Dsg2 or Dsc2 and performing liver perfusion experiments, we demonstrated that expression levels of desmosomal adhesion molecules in the liver tissue have no impact on the early steps of metastasis formation. Finally, our results indicate that the cluster size of CTCs is a relevant factor contributing to the initial attachment of cancer cells to the liver.

Our study adds a model to the toolbox of experimental approaches to study metastasis formation bridging in vivo animal models and in vitro liver models. This ex vivo model has the advantage to individually access the very first steps of metastatic seeding via the physiological dissemination route in situ under controlled conditions. In contrast to the most commonly used murine in vivo metastasis models with primary tumor seeding by implantation of tumor cells (allograft vs. xenograft), direct injection of tumor cells e.g. into the spleen or portal vein, or with genetic engineering of tumor formation, this post mortem liver perfusion model offers a refined approach with a lower burden for the animal and can circumvent some disadvantages of certain in vivo models such as the use of immunocompromised animals or non-representative genetic modifications^29–31^. Beside in vivo experiments, in vitro liver tissue-engineering approaches (e.g. LiverChip or PEARL) are applied to investigate specifically the initial phase of tumor cell attachment. To recapitulate liver tissue structure, hepatocytes are seeded on scaffolds and cultivated under flow with addition of tumor cells to the perfusion solution. In contrast to the presented ex vivo perfusion, the major limitation of the in vitro approaches is the low biological transferability, as they only partially recapitulate the complex liver cell architecture (sinusoids, space of Disse, portal fields, central vein), which is relevant for tumor cell attachment^32–34^. While the application of cultivated liver slices overcomes this disadvantage, the approach does not reproduce the physiological way of tumor cells approaching the liver through the portal vein, as tumor cells are directly seeded on the sections^35^.

In conclusion, the here established ex vivo liver perfusion can minimize animal burden with seeding of tumor cells to intact organs with physiological liver histology and can thus overcome drawbacks of in vitro and in vivo models to study early liver metastasis formation. From other fields, ex vivo organ perfusion of the liver is well established to evaluate functional parameters^36–38^, and was here adapted to investigate early tumor cell metastasis attachment.

We tested the murine ex vivo model using the pancreatic cancer cell line AsPC-1 with and without expression of the core desmosomal adhesion molecule DSG2. Our data demonstrate that expression of DSG2, presumably via adhesion-dependent cell cluster formation, is important for initial deposition of AsPC-1 cells in liver tissue. Cells depleted for DSG2, correlating with reduced cluster size of these cells, were less retained within the perfused liver. In line with this, desmosomal transcripts were shown to be strongly upregulated in K14-expressing CTC clusters in breast cancer models, suggesting that homotypic CTC clusters rely on desmosomal adhesion^39^. This is further supported in studies in breast cancer, in which CTC clusters were shown to depend on the presence of the desmosomal adapter protein PG^20^. However, studies showing that other adhesion molecules such as CD44^40^, or ICAM-1^41^ contribute to the formation and maintenance of CTC aggregates suggest this effect to be more dependent on the contribution of desmosomal molecules to cell–cell adhesion in general than DSG2 expression specifically. In line with this, it is now well established that homotypic CTC clusters and more specifically their potential to form metastases depend on intercellular adhesion molecules in several cancer types^42^.

This is further supported by our finding of a decreased rate of tumor cell attachment in response to artificial reduction of the tumor cluster size. Similar to CTC clusters of breast and prostate cancer, which show higher pathogenicity compared to single CTCs^42^ our study suggests that this is also relevant for pancreatic cancer cells. This higher deposition is presumably related to the physical size of a CTC cluster, which should have a higher propensity to get stuck in the liver sinusoids than solitary CTCs. Those clusters should than also be better in withstanding the relatively high flow velocity in hepatic sinusoids^43^. Additionally, once the CTCs are stuck in the liver, local invasion and proliferation of the newly formed metastasis may benefit from an initially higher number of tumor cells. Due to the relatively short time frame of the observation period in this study, we did not further investigate differences in local invasion and proliferation in AsPC-1 cells deposited in the liver. Nevertheless, in vivo studies with an injection of AsPC-1 cells via the tail vein showed a higher metastatic burden in correlation with DSG2 expression and larger cluster size in the depending capillary bed in the lung. It is possible that, independent of the initial differences in the deposition, the presence of DSG2 may contribute to alterations in the final steps of metastasis formation leading to differential metastatic load. This is relevant, as adhesion molecules, such as the classical cadherin E-cadherin, were shown to differentially contribute to different steps of the metastatic cascade^44^. Along similar lines, by using the same cell lines as in the current study, we have shown that loss of DSG2 confers a pro-migratory and pro-invasive behavior^17^. Thus, DSG2 would potentially inhibit metastasis at this early step of the metastatic cascade by preventing invasion and dissociation from the primary tumor. However, with regard to CTC transmission in the blood stream, our ex vivo and in vivo data support, together with the available body of literature and clinical data, the concept that increased cell–cell adhesion of CTC’s supports liver deposition and metastasis formation. This suggests that, by investigating the different steps individually, also for desmosomal molecules a differential contribution to the multiple aspects of the metastatic cascade is likely.

Interestingly, these results were independent from the levels of Dsg2 in hepatocytes of the recipient livers. The deposition of AsPC-1 cells was not different when hepatocyte-specific Dsg2 or Dsc2 KO livers were perfused. This indicates that initial adhesion of tumor cells in the hepatic tissue is not mediated via binding to desmosomal adhesion molecules, suggesting that desmosomes are more relevant for cluster morphology than attachment to hepatic cells. Hepatocytes are the only cell type in the liver expressing desmosomes. However, cells in the blood stream are primarily in contact with the sinus endothelium, which, while far from tight, still forms a physical barrier to the space of Disse and the hepatocytes. Although we observed AsPC-1 cells in contact with hepatocytes, it is possible that the short contact and thus insufficient interaction time of tumor cell surface proteins with the hepatic cell surface in our observation period contributes to the lack of an effect of hepatocyte levels of desmosomal cadherins. Alternatively, loss of desmosomal adhesion molecules may only become relevant in in vivo setups, in which the entire metastatic cascade is modeled. It would be important to test whether the formation of the premetastatic niche, which represents a prometastatic environment primed by the primary tumor^45^, shows differences in composition and function dependent on the presence of Dsc2 or Dsg2 in the liver.

Together, this study contributes to a better understanding of the role of desmosomes and cell–cell adhesion during the metastatic process, which seems to be differential during the seperate steps. Our study demonstrates a direct correlation between the cluster size of tumor cells and the rate of early attachment of metastatic cells to the liver. Here, desmosomal adhesion seems to be a relevant factor contributing to the formation of larger cell cluster. This is relevant with respect to therapeutic strategies, as previous studies in cardiac and skin disease models prove direct modulation of desmosomal adhesion as a promising approach to modify cellular connection and behavior^46,47^.

## Methods

### Cell lines and culture

The pancreatic ductal adenocarcinoma cell line AsPC-1, which is derived from cells from the ascites of a patient with pancreatic cancer after implantation into nude mouse xenografts^48^, was cultured in a humidified atmosphere containing 5% CO_2_ at 37 °C in RPMI 1640 Medium + GlutaMAX (Gibco) supplemented with 10% fetal bovine serum (S0615, 10 Merck, Darmstadt, Germany), 50 U/mL penicillin (VWR International) and 50 µg/mL streptomycin sulfate (VWR International). The culture medium was changed every 3 days. Once the cells reached 80–90% confluency, they were detached with 0.06% trypsin (T/3760/48, Fisher Scientific) in PBS with 1% EDTA (20309.296, VWR) and transferred to a new dish using a split ratio of 1:1.5–1:3. The DSG2 KO clones were generated using the CRISPR/Cas9 technique. The DSG2 KO was confirmed using next-generation sequencing and Western blot analysis. Infection with mycoplasma was excluded by repetitive PCR evaluations. AsPC-1 cells were routinely authenticated by Short Tandem Repeat profiling. For tracing of AsPC1 cells in the xerograph model, cell were lipotransfected with pEGFP-N1 (Addgene, Cat 6085-1) and selected by G418.

### Generation of liver-specific Dsg2 and Dsc2 KO mouse model

Mouse perfusion experiments were carried out according to the protocol approved by the Cantonal Veterinary Office of Basel-Stadt (License number 2973_32878) and complied with the ARRIVE guidelines. All mice were housed under specific pathogen-free conditions with standard chow and bedding with 12 h day/night cycle according to institutional guidelines. Animals of both sexes were applied without bias. To generate hepatocyte-specific Dsg2 or Dsc2 KO mice (Dsg2 KO; Dsc2 KO), B6.129 *Dsg2*^*tm1Mdcb*^ or *Dsc2*^*tm1Mdcb*^^49^, (provided by the Max-Delbrück-Centrum für Molekulare Medizin in der Helmholtz-Gemeinschaft, Arnd Heuser) which homozygously express loxP sites flanking exon 2 of the *Dsg2* or *Dsc2* gene, respectively, were crossed with B6.Cg-Speer6-ps1^Tg(Alb-cre)21Mgn^/J (Strain#: 003574, The Jackson Laboratory), which heterozygously express the recombinase Cre under the control of the hepatocyte-specific albumin promotor, to generate liver-specific KO animals. These F1 animals were bred with the parental line to generate homozygous floxed KO animals. For genotyping of the animals, DNA was extracted from biopsies in 25 mmol/L NaOH and 0.2 mmol/L EDTA at 98 °C for 1 h and neutralized with 40 mmol/L Tris pH 5.5. PCR was performed using GoTaq G2 (M7845, Promega, Madison, WI, USA) according to manufacturer’s instructions with the primers Dsg2 for CCAGAGGAAACAACCTGGAA and rev GCACAGGACTCAGGATTGGT, Dsc2 for CCCTCCCAGTCAGTGAAGTTA and rev TTTGATACCCAGCACACCTTT, or Alb-Cre for CCAGGCTAAGTGCCTTCTCTACA, and rev AATGCTTCTGTCCGTTTGCCGGT, which span the floxed region of *Dsg2* or *Dsc2* or the Cre transgene. Respective KO of experimental animals was confirmed by Western blot analysis.

### Cell preparation for liver perfusion experiments

AsPC-1 cells were seeded on a 145 mm petri dish and grown to confluency for 3 days. After washing with PBS, the cells were incubated with dispase II (> 2.4 U/mL, D4693, Sigma-Aldrich) in HBSS (H8264, Sigma-Aldrich) for 30 min at 37 °C. After detachment, the cells were resuspended in PBS containing 1% EDTA (20,309.296, VWR) to inhibit dispase activity and detach cells. To eliminate excess dispase and EDTA, the cell suspension was centrifuged for 3 min at 300 G and resuspended in PBS. To identify AsPC-1 cells in later analysis, cells were stained with CellTrace Far Red (C34572, Thermo Fisher Scientific) at a concentration of 1 µM and incubated for 10 min at 37 °C in the dark. After incubation, staining reaction was quenched by addition of RPMI 1640 medium without l-Glutamine (1-41F51-I, BioConcept). After centrifugation for 3 min at 300 G, the stained AsPC-1 cells were resuspended in sterile Krebs–Henseleit buffer (118 mM NaCl, 4.7 mM KCl, 1.2 mM MgSO_4_, 1.25 mM CaCl_2_, 1.2 mM KH_2_PO_4_, and 25 mM NaHCO_3_ in distilled water) and strained through a 40 μm cell strainer (Falcon). Cells were resuspended in Krebs–Henseleit buffer with a concentration of 20,000 cells per ml. Images of the counting chambers were taken with an SLR camera (EOS 800D, Canon, Tokyo, Japan) for cluster analysis of the input cells. In total 1,000,000 cells in Krebs–Henseleit buffer were transferred to a 50 mL syringe, which was rinsed beforehand with fetal bovine serum (S0615, 10 Merck, Darmstadt, Germany) to reduce adhesion of AsPC-1 cells to the enclosure of the syringe. The remaining AsPC-1 input cells were stored at 4 °C until further analysis via flow cytometry.

To investigate the importance of the cluster size, AsPC-1 control cells were seeded and detached from the dish by incubation with (1) dispase II (> 2.4 U/mL, D4693, Sigma-Aldrich) in HBSS (H8264, Sigma-Aldrich) for 30 min or (2) 0.06% trypsin (T/3760/48, Fisher Scientific) in PBS with 1% EDTA (20309.296, VWR) for 20 min. In comparison to the cell preparation process stated above, cells were additionally filtered through a 40 μm cell strainer (Falcon) twice and fixed in 4% paraformaldehyde (P/0840/53, Thermo Fisher Scientific) in PBS to increase number of single cells and prevent re-clustering. The cell suspension was centrifuged for 3 min at 300 G and resuspended in Krebs–Henseleit buffer to remove paraformaldehyde. All following steps, including the mouse dissection, liver perfusion, and analysis workup, were performed accordingly.

### Mouse dissection for liver perfusion experiments

Mice were sacrificed by an overdose of pentobarbital according to guidelines of the Cantonal Veterinary Office of Basel-Stadt and the University of Basel. Dissection was performed under a binocular stereo microscope (SZX2, Olympus, Shinjuku, Japan). After loss of pain reflexes, a midline laparotomy and lateral thoracotomy were performed. 500 μL heparin (1000 U/mL, 11483277, Thermo Fisher Scientific) in PBS was injected into the beating left ventricle to avoid blood clotting. To block retrograde flow, the abdominal part of the inferior vena cava was ligated. For perfusion, the mouse was transferred into an organ perfusion set-up (UP-100, Harvard Apparatus) with a moist chamber (Type 834/8), heat exchanger (TC120-ST5), peristaltic pump (ISM 827), bubble trap, pressure transducer (APT300), and a monitoring system setup using the PLUGSYS Amplifier System (TAM-D type 705/2 and SCP type 704) to measure the portal venous flow and pressure. The vena portae was exposed and a cannula (inner diameter 0.7 mm, outer diameter 1.0 mm) was inserted into the vein via a small incision followed by cannula attachment and ligation. The same procedure was repeated for the suprahepatic inferior vena cava with another cannula (inner diameter 1.0 mm, outer diameter 1.3 mm) via incision of the right atrium. Once the cannulas were fixed, the perfusion with oxygenated (carbon dioxide oxygen mixture, 95% O_2_, 5% CO_2_) Krebs–Henseleit buffer (118 mM NaCl, 4.7 mM KCl, 1.2 mM MgSO_4_,1.25 mM CaCl_2_, 1.2 mM KH_2_PO_4_, and 25 mM NaHCO_3_ in distilled water) was started at a set portal venous pressure of 5 mmHg with automatically adapting flow. The initial outflow via the cannula in the inferior vena cava was discharged. With the defined portal venous pressure of 5 mmHg, the portal venous flow establish after about 10 min at around 5–10 mL per minute was achieved. The perfusion was continued for in total 60 min to stabilize the flow prior to cell injection.

### Liver perfusion experiments

Once the flow has stabilized, 1,000,000 AsPC-1 control or DSG2 KO cells, resuspended in Krebs–Henseleit buffer in a 50 mL syringe were perfused into the liver via the hepatic portal vein using a syringe pump (Pump 11 Elite, Harvard Apparatus) with a flow ratio of 1:10 of the established portal flow at 5 mmHg for approximately 60 min. This resulted in a final concentration of 2000 tumor cells per perfused milliliter total buffer. After 30 min, AsPC-1 output cells were collected from the outflow of the liver via the cannula in the inferior vena cava and fixed in 1% paraformaldehyde (P/0840/53, Thermo Fisher Scientific) in PBS. To increase the concentration of AsPC-1 output cells, the cells were centrifuged for 3 min at 300 G, and supernatant was reduced to 1 mL. Once all the AsPC-1 cells were injected, the liver was perfused with oxygenated Krebs–Henseleit buffer for additional 30 min. To determine the rate of perfused liver cells, IRDye 800CW NHS Ester (P/N 929-70020, Li-Cor) diluted in Krebs–Henseleit buffer to a concentration of 5 µg/mL was perfused for 5 min with the established flow rate to label all perfused liver cells. A sample of the output was collected at the end of the experiment via the cannula in the inferior vena cava and an ALT assay was performed to assess the perfusion-related damage to liver cells. Criteria for sufficient perfusion were defined as a perfusion rate of more than 90%, a portal venous flow between 5 and 10 mL per minute, and an ALT activity of less than 2 mU/mL.

### Liver digestion and flow cytometry analysis

After perfusion, the common bile duct was clamped and a cholecystectomy was performed to prevent excessive autofluorescence of the bile in later immunostainings. A lobectomy of the right lateral liver lobe was performed and tissue was fixed in 4% paraformaldehyde (P/0840/53, Thermo Fisher Scientific) in PBS for cryosections and immunostainings. The rest of the liver was removed from the mouse situs, transferred to a petri dish, cut into small pieces, and incubated in 10 mL 0.06% trypsin (T/3760/48, Fisher Scientific) in PBS supplemented with 1% EDTA (20,309.296, VWR) at 37 °C on a roller for 10 min. After incubation, the liver cells were mashed through a 70 μm cell strainer (Falcon). To stop digestion, cells were washed with FACS buffer (2% fetal bovine serum and 0.1% sodium azide in PBS), centrifuged for 3 min at 300 G, resuspended in FACS buffer and fixed in 2% paraformaldehyde. To ensure a single cell suspension, samples of dissociated livers were vortexed and filtered through a 40 μm cell strainer (Falcon) before flow cytometry. The samples were analyzed with a CytoFLEX flow cytometer (Beckman Coulter Life Sciences). The fluorescence channel “APC” (excitation laser 640 nm, filter 660/20) was used for detection of CellTrace Far Red (C34572, Thermo Fisher Scientific) stained AsPC-1 cells, and the channel “APC-A750” (excitation laser 640 nm, filter 780/60) to distinguish IRDye 800CW NHS Ester (P/N 929-70020, Li-Cor) labeled liver cells. Results were analyzed using FlowJo (version 10.8.1, Becton Dickinson & Company). See Supplementary Fig. S1 for respective gating strategies.

### ALT assay

Serum samples from the outflow of the mouse livers were obtained via the cannula in the suprahepatic inferior vena cava. The enzymatic activity of the ALT was determined with an ALT Activity Assay Kit (MAK052, Sigma-Aldrich) according to the manufacturer’s instructions by measuring fluorometric signal changes proportional to the amount of pyruvate generated by the presence of ALT enzyme. Krebs–Henseleit buffer served as background control.

### Immunostaining

Tissue samples were acquired from a lobectomy of the right lateral liver lobe at the end of the liver perfusion experiments and were fixed in 4% paraformaldehyde (P/0840/53, Thermo Fisher Scientific) in PBS. Samples were first incubated in 15% sucrose in PBS, followed by incubation in 30% sucrose in PBS at 4 °C overnight. The tissue was embedded in embedding medium (12% mowiol 4–88, 5% sorbitol, 0.5% bovine serum albumin, and 0.025% sodium azide) and frozen in isopentane at − 60 °C. The entire lobe was cut transversally into 10 µm thick tissue sections with a Cryo Star NX70 cryostat (Thermo Fisher Scientific) and transferred to SuperFrost plus glass slides (Thermo Fisher Scientific). For immunostaining, the sections were air-dried at 37 °C for 30 min, permeabilized in 0.2% triton X-100 (Thermo Fisher Scientific) in PBS for 15 min and blocked with 3% bovine serum albumin and 0.12% normal goat serum in PBS for 60 min. As a primary antibody to label hepatic vessels, rabbit anti-laminin (L8271, Sigma-Aldrich) was incubated overnight in PBS at 4 °C. As secondary antibody, goat anti-rabbit coupled to Alexa Fluor 488 in PBS (Thermo Fisher Scientific) were incubated for 60 min at room temperature. DAPI working solution (1 mg/mL, D9542, Sigma-Aldrich, in distilled water) was added for 10 min to stain cell nuclei. The stained sections were mounted with Fluoromount Aqueous Mounting Medium (Sigma-Aldrich).

### Image acquisition and analysis of liver sections

For image acquisition, an Eclipse Ni-E microscope with a 10× Plan Apo λ objective (both Nikon), captured by a Prime 95B camera (Teledyne Photometrics), attached to a slide loader (Prior Scientific) was used. Per mouse, 2 consecutive liver sections, respectively, from the top, middle, and bottom level of the lobe (min. 0.5 mm distance between the levels) were acquired and analyzed. The determination of liver tissue area was conducted via pixel classification analysis of autofluorescence signals emitted by the liver and the number of cells per slide were determined using the Watershed Cell Detection method, detecting DAPI-stained cell nuclei. CellTrace Far Red (C34572, Thermo Fisher Scientific) labeled AsPC-1 cells were identified and differentiated from liver cells by a set threshold for the mean and max cytoplasmatic signal of previously detected DAPI-positive cells. For the discrimination of a single AsPC-1 cell and AsPC-1 cells in a cluster, a cell–cell distance of 50 µm and a minimal number of two cells were required to fulfill the criteria of a cell cluster. Image analysis was done with QuPath (QuPath developers, The University of 18 Edinburgh, UK, version 0.2.3).

### Western blot analysis

Samples were obtained from confluent AsPC-1 cells or liver from the right lateral lobe. The cell samples were washed with PBS and lysed in SDS lysis buffer (25 mM HEPES, 2 mM EDTA, 25 mM NaF, and 1% SDS in distilled water) supplemented 1:1 with the protease inhibitor cocktail cOmplete (11697498001, Sigma-Aldrich). Tissue samples were lysed in the same buffer with steal beads using a tissue bead beating homogenizer (FastPrep-24 5G, MP Biomedicals, Santa Ana, CA, USA) and subsequently cleared by centrifugation. Lysates were sonicated and the total protein amount was determined with a BCA protein assay kit (Thermo Fisher Scientific) as stated by the manufacturer’s instructions. Lysates were denaturized for 5 min at 95 °C in Lämmli buffer, followed by gel electrophoresis and wet blotting on nitrocellulose membranes (Novex, Thermo Fisher Scientific) according to standard procedures. The membranes were blocked using Odyssey blocking buffer (Li-Cor) diluted 1:1 in TBS for 60 min at room temperature. The following primary antibodies were diluted in TBS 1:1 with Odyssey blocking buffer (Li-Cor) containing 0.2% tween 20 (BP337-500, Thermo Fisher Scientific) and incubated overnight at 4 °C: Mouse anti-DSG1/2 (61002, PROGEN Biotechnik, Heidelberg, Germany), mouse anti-DSC2/3 (15909897, Thermo Fisher Scientific), mouse anti-DP (61003, PROGEN Biotechnik), mouse anti-PKP2 (651101, PROGEN Biotechnik), mouse anti-PG (61005, PROGEN Biotechnik), mouse anti-E-Cadherin (610181, BD Bioscience), mouse anti-β-catenin (610154, BD Bioscience), mouse anti-N-Cadherin (610921, BD Biosciences, Franklin Lakes, NJ, 24 USA) and rabbit anti-GAPDH (10494-1-AP, Proteintech, Rosemont, IL, USA). As corresponding secondary antibodies, goat anti-mouse 800CW (925-32210, Li-Cor) or goat anti-rabbit 680RD (925-68071, Li-Cor) were diluted in TBS containing 0.1% tween 20 and incubated for 60 min at room temperature. The Odyssey FC imaging system (Li-Cor) was applied to detect protein bands. After normalization to the loading control, band density was quantified using ImageStudio (version 5.2, Li-Cor).

### RNA isolation and qPCR

Samples were obtained from mouse livers and washed in ice-cold PBS. Tissue homogenization was conducted via the FastPrep-24 5G bead beating grinder (MP Biomedicals, Santa Ana, CA, USA) using 2.8 mm stainless steel beads (Sigma-Aldrich) according to manufacturer’s protocol with subsequent centrifugation to clear the lysate. RNA was isolated via the Direct-zol RNA MiniPrep kit including Zymo-Spin II and DNAse restriction step (R2050, Zymo research, Irvine, CA, USA). Quantity and quality of RNA was determined by Nanodrop 1000 Spectrophotometer (Thermo Fisher Scientific). Up to 1 µg of isolated RNA was used for reverse transcription with SuperScript III (Thermo Fisher Scientific). Quantitative real time PCR was performed using Power SYBR Green PCR Master Mix (Thermo Fisher Scientific). The following primer pairs were applied: m*Dsg2*_for: TCTACCTAAATAAAGACACGGGG; m*Dsg2*_rev: TAGCTGCTGTGTTCCTCTCT; m*Dsc2*_for: CCTGTTGACCCTTGCGATCCT; m*Dsc2*_rev: CAGGTAGTTCAGAGGGGACCT; m*Gapdh*_for: CCCACTCTTCCACCTTCGAT; m*Gapdh*_rev: AGTTGGGATAGGGCCTCTCTT. As reference, the Ct value of Gapdh of the respective sample was used.

### Dissociation assay

For dissociation experiments to determine cell–cell adhesion, AsPC-1 cells were seeded on TC-treated plastic 24-well plates and grown to confluence. Cell monolayers were washed with HBSS and incubated with dissociation buffer (dispase II 2.5 U/mL, Sigma-Aldrich, D4693 in HBSS) at 37 °C. After detachment, defined mechanical shear stress was applied to the floating cell monolayers by pipetting using an electrical pipette (Eppendorf, Hamburg, Germany). The total number of resulting fragments per well was determined using a binocular stereo microscope (SZX2, Olympus). For documentation, images of the wells were acquired with a SLR camera (EOS 800D, Canon).

### Murine xenografts

All in vivo animal studies were approved by the Institutional Animal Care and Use Committee of CINVESTAV-IPN and performed according to the ethical institutional guidelines and the ones established by Mexican authorities (protocol 0227-16; 08/12/16). Animals were kept under pathogen-free conditions in a barrier-type facility at CINVESTAV-IPN (Mexico City, Mexico). For lung metastasis assays, 6–8-week-old mice (NOD.Cg-PrkdcscidIl2rgtm1Wjl/SzJ) were injected i.v. with 0.5 × 10^6^ AsPC-1 GFP^+^ Ctrl or DSG2 KO cells in 100 µL of saline solution into the tail vein. Animals were monitored twice weekly and were sacrificed after 8 weeks or earlier when the humane endpoint was reached. After termination, lungs were collected and digested for 30 min using 5 mg/mL Collagenase A + 1 ug/mL DNase I, the suspension was washed and passed through a 70 µm strainer and the obtained cell suspension prepared for flow cytometry. Lung cell suspensions were analysed on a FACS Canto II cytometer, and percentage of GFP^+^ (AsPC1) cells was analyzed using FlowJo X software (BD Biosciences, Franklin Lakes, NJ).

### Statistic and data analysis

Figures were compiled with Adobe InDesign 2022 (version 17.4), Adobe Illustrator 2022 (version 26.5), and Adobe Photoshop 2022 (version 23.5.1) (all Adobe, San José, CA). Statistical analysis was performed with Prism 8 (GraphPad, La Jolla, CA). For comparison of 2 or multiple groups, distribution of data was analyzed by a Shapiro–Wilk normality test, and group variances were analyzed with the *F*-test. According to the results of these tests, a parametric or nonparametric test with or without Welch correction for unequal variances was applied. The statistical test used to compare the respective data sets is described in the corresponding figure legend. Alpha level for all tests is 0.05. Statistical significance was assumed at *P* < 0.05. Unless otherwise stated, data are presented as dot blot, with each dot representing the mean of the respective technical replicates of 1 biological replicate. Each animal or independent seeding of cells was taken as biological replicate, or as indicated in the legend. The mean value of these dots is shown as bar diagram ± s.d. Grammarly for Windows (version 1.0.41.861) was used for text correction purposes. No text content was written by artificial intelligence.

### Supplementary Information

Supplementary Figures.

## Data Availability

The data supporting the findings of this study are available from the corresponding authors upon request.
