# Supplementary material for: Adhesion of pancreatic tumor cell clusters by desmosomal molecules enhances early liver metastases formation
Source: Sci Rep. 2024 Aug 6;14:18189. doi: 10.1038/s41598-024-68493-6 (PMC11303515; doi:10.1038/s41598-024-68493-6)
Supplement: Supplementary file 1 — Supplementary Figures. [file 41598_2024_68493_MOESM1_ESM.pdf]

SUPPLEMENTARY INFORMATION

Adhesion of pancreatic tumor cell clusters by desmosomal molecules enhances early liver metastases formation

Niclas Dietrich[1], Ramon Castellanos-Martinez[1,2], Julia Kemmling[3], Arnd Heuser[4], Michael Schnoor[2], Camilla Schinner[1,4, †, *], Volker Spindler[1,3 †, *]

[1] Department of Biomedicine, University of Basel, Basel, Switzerland

[2] Department of Molecular Biomedicine, CINVESTAV-IPN, Mexico City, Mexico

[3] Institute of Anatomy and Experimental Morphology, University Medical Center Hamburg Eppendorf, Hamburg, Germany

[4] Animal Phenotyping Platform, Max-Delbrück Center for Molecular Medicine in the Helmholtz Association, Berlin, Germany

[5] Hannover Medical School, Institute of Applied and Functional Anatomy, Hannover, Germany

[†] These authors contributed equally.

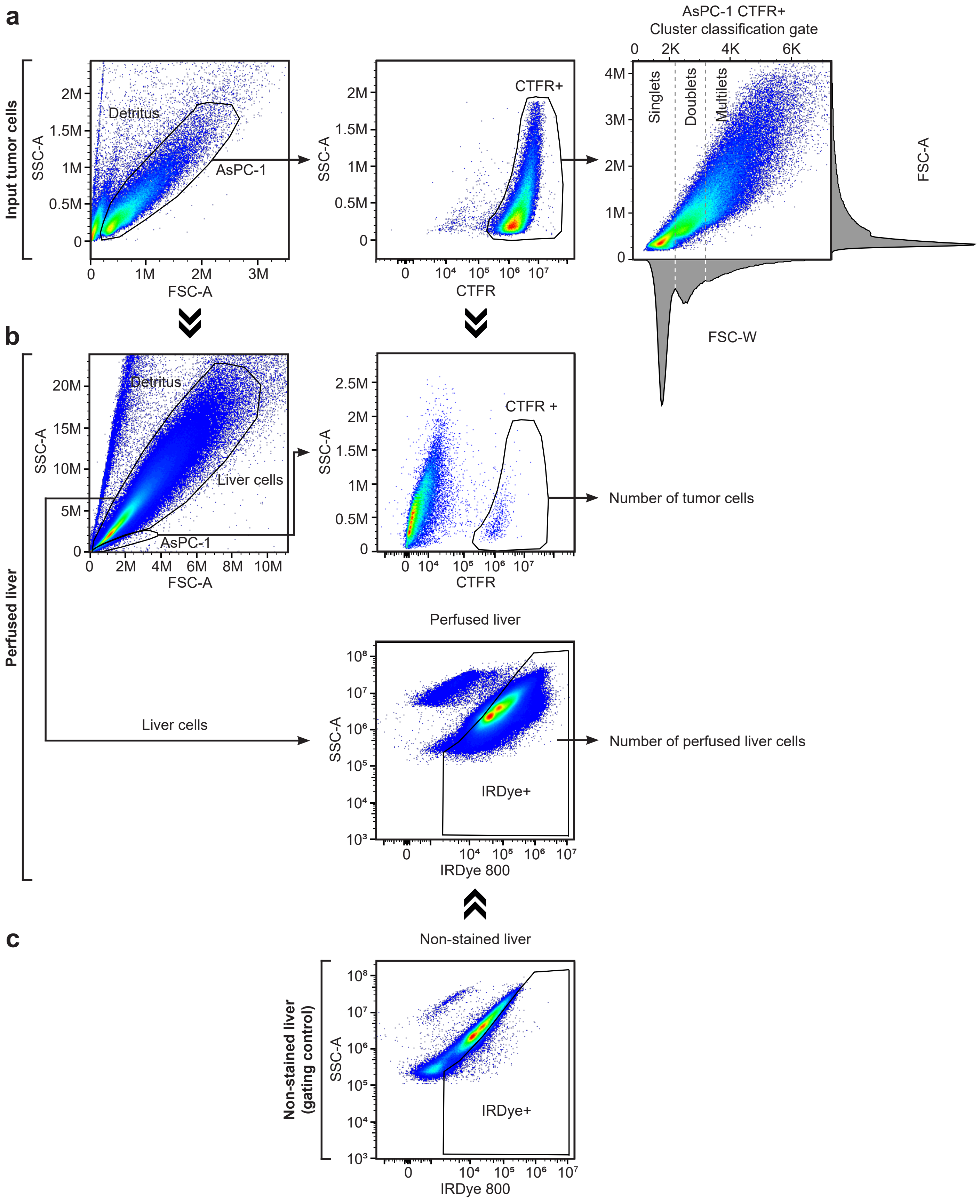

Supplementary Figure 1.

Supplementary Figure S1. Gating strategy of flow cytometry analysis

a. Strategy to set gates to determine perfused AsPC-1 cells: In data from flow cytometry analysis of input AsPC-1 cells, a cell size gate (FSC vs SSC) was set to eliminate cell detritus. From this population, CellTrace Far Red (CTFR) stained AsPC-1 tumor cells were gated as a CTFR-positive population. To analyse clustering of these cells, the forward scatter width plot (FSC-W) was used to classify the AsPC-1 cells in singlets, doublets and multilets as indicated. Adjacent histograms indicate the relative frequency. **b**. Strategy to set gates to determine attached tumor cells per perfused liver cells: To depict the population of liver cells, the AsPC-1 cell size gate described in (a) was overlayed on the perfused mouse liver sample and the larger and more complex liver cells were gated in addition. For detection of the stained AsPC-1 cells in the liver sample, the same CellTrace Far Red gate described in (a) was applied. **c.** To determine the percentage of perfused liver cells, IRDye 800CW NHS Ester labeled cells were detected. For setting of the respective gates, a non-stained control liver sample was analyzed and a “non-perfused” cell gate was set to include 95% of the non-stained cells. The IRDye positive “perfused” cells gate was adjusted accordingly to include 5% of the non-stained cells.

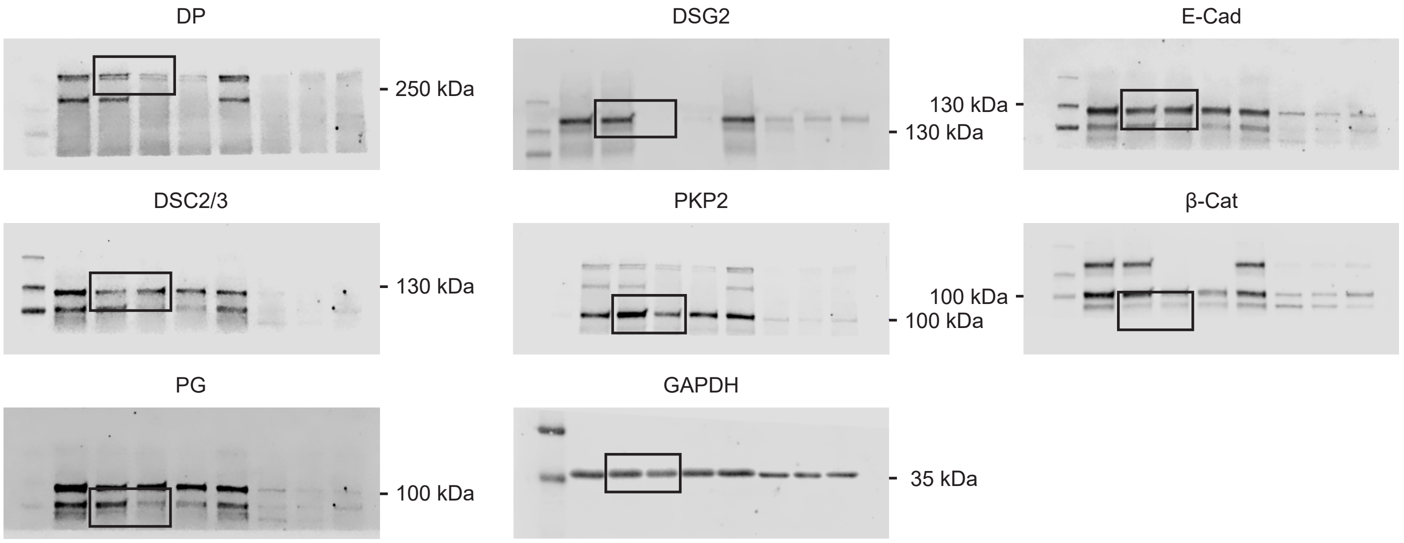

Figure 2a uncropped blots

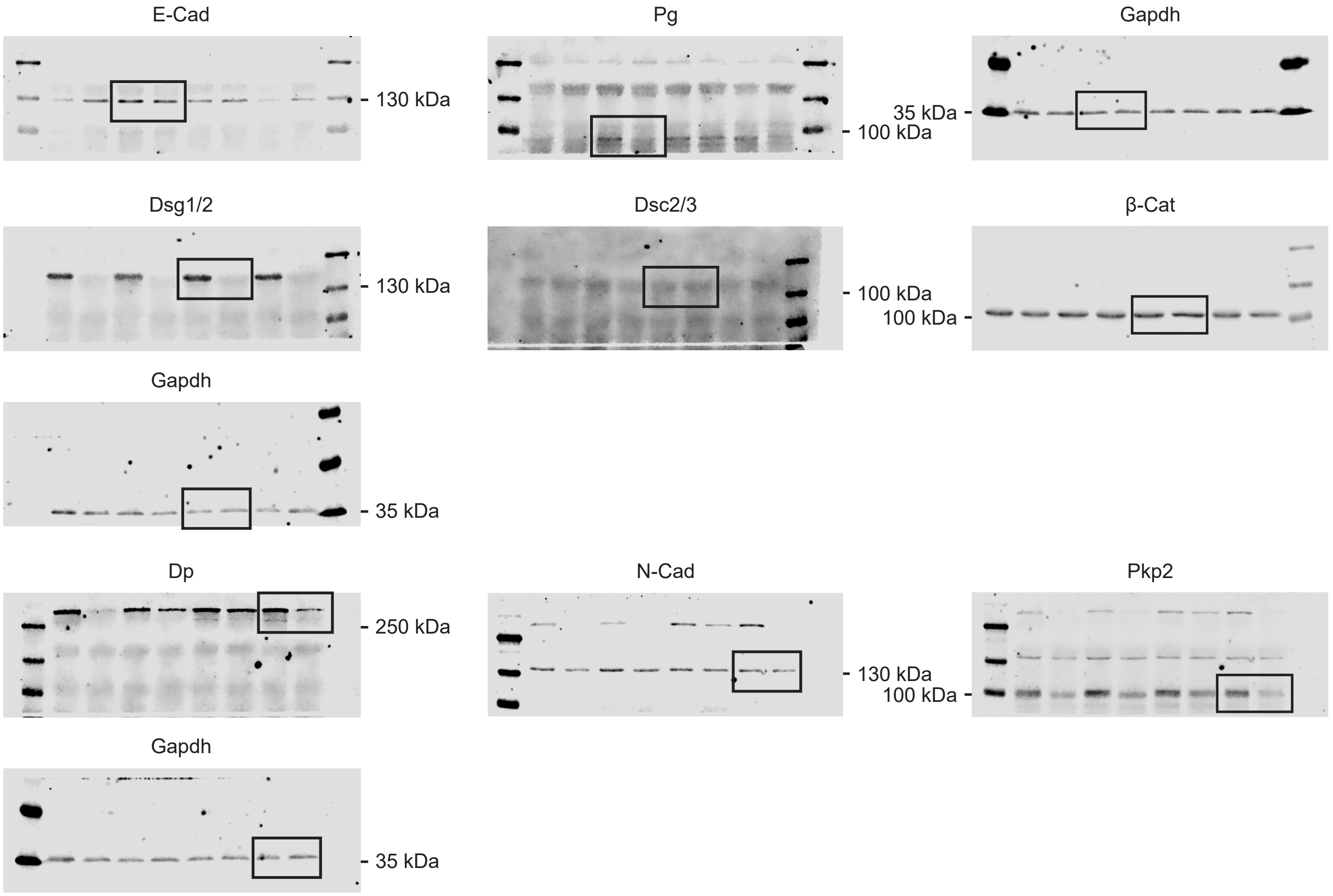

Figure 3a uncropped blots Dsg2 KO mouse model

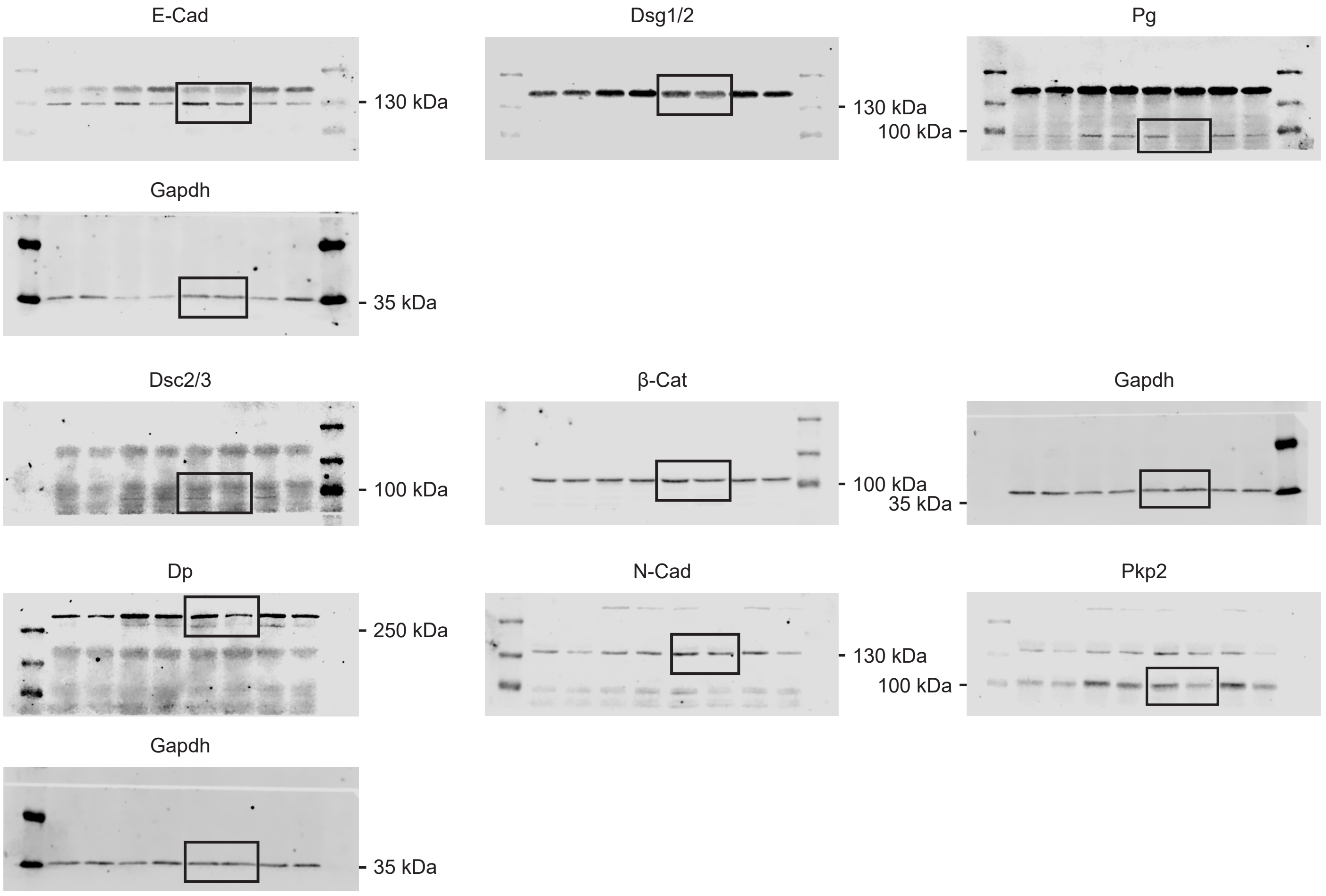

Figure 3a uncropped blots Dsc2 KO mouse model
